# A system biology approach based on metabolic biomarkers and protein–protein interactions for identifying pathways underlying schizophrenia and bipolar disorder

**DOI:** 10.1038/s41598-021-93653-3

**Published:** 2021-07-14

**Authors:** Md. Altaf-Ul-Amin, Kazuhisa Hirose, João V. Nani, Lucas C. Porta, Ljubica Tasic, Shaikh Farhad Hossain, Ming Huang, Naoaki Ono, Mirian A. F. Hayashi, Shigehiko Kanaya

**Affiliations:** 1 Nara Institute of Science and Technology, Ikoma, Nara 630-0192 Japan; 2 Department of Pharmacology, Escola Paulista de Medicina (EPM), Universidade Federal de São Paulo (UNIFESP), São Paulo, SP Brazil; 3 National Institute for Translational Medicine (INCT-TM, CNPq/FAPESP/CAPES), Ribeirão Preto, Brazil; 4 Chemical Biology Laboratory, Department of Organic Chemistry, Institute of Chemistry, Universidade Estadual de Campinas (Unicamp), Campinas, SP Brazil

**Keywords:** Computational biology and bioinformatics, Molecular biology, Neuroscience, Systems biology, Biomarkers, Diseases

## Abstract

Mental disorders (MDs), including schizophrenia (SCZ) and bipolar disorder (BD), have attracted special attention from scientists due to their high prevalence and significantly debilitating clinical features. The diagnosis of MDs is still essentially based on clinical interviews, and intensive efforts to introduce biochemical based diagnostic methods have faced several difficulties for implementation in clinics, due to the complexity and still limited knowledge in MDs. In this context, aiming for improving the knowledge in etiology and pathophysiology, many authors have reported several alterations in metabolites in MDs and other brain diseases. After potentially fishing all metabolite biomarkers reported up to now for SCZ and BD, we investigated here the proteins related to these metabolites in order to construct a protein–protein interaction (PPI) network associated with these diseases. We determined the statistically significant clusters in this PPI network and, based on these clusters, we identified 28 significant pathways for SCZ and BDs that essentially compose three groups representing three major systems, namely stress response, energy and neuron systems. By characterizing new pathways with potential to innovate the diagnosis and treatment of psychiatric diseases, the present data may also contribute to the proposal of new intervention for the treatment of still unmet aspects in MDs.

## Introduction

Schizophrenia (SCZ) and bipolar disorder (BD) are severe debilitating mental disorders (MDs), as both are associated with cognitive impairments, and altered behavior, mood and perceptions. Together, these MDs affect around 100 million people worldwide, irrespective of nationality, ethnic origin, or socioeconomic status^1^. SCZ is characterized by a set of positive and negative symptoms and, according to the 5th version of the Diagnostic and Statistical Manual of Mental Disorders (DSM-V), at least two or more of these symptoms need to be present in an individual for the diagnosis. In turn, BD is a chronic mood disorder often characterized by the fluctuations between mania and depressive episodes, and due to the complex mood alterations, the clinical misdiagnosis in BD is common^2^. In addition, other MDs, such as major depressive disorders, also shares several common symptoms and specific endophenotypes with SCZ and BD^3–5^. In fact, pathophysiology of all these MDs are mainly centered in the hypothesis of alterations in dopamine homeostasis and signaling^6,7^, while gamma-aminobutyric acid (GABA) is the principal inhibitory neurotransmitter found altered in SCZ^8–11^. SCZ and BD share several symptoms and, up to a certain degree, also share similar pharmacological interventions. For instance, the second-generation antipsychotics (SGAs) were primarily developed for the treatment of the positive and negative symptoms of SCZ^12^, while they are also currently used as an alternative to lithium for the suppression of the main symptoms in BD^13^.

SCZ and BD are highly polygenic diseases, with many associated genetic variants with small effects, as demonstrated by several genome-wide association studies (GWAS)^14–17^. Moreover, several genetic-based studies have shown a limited contribution to support the diagnosis and/or the characterization of pathways underlying these major MDs, due to the well-recognized pleiotropic features and small size effect of each gene^18,19^. Therefore, none of genetic studies was adequate to reveal the mechanisms underlying the etiology and/or pathophysiology of SCZ and/or BD.

In this context, proteomics and metabolomics are now recognized as valuable tools for the characterization of any disease. Although proteins are the functional actors that are responsible for the generation or catabolism of all metabolites, identification of metabolic processes characteristically active or inactive in any specific healthy and/or pathological conditions may uncover pathways important for the understanding of disease mechanisms. Interestingly, the separation of SCZ and healthy control (HC) individuals, by metabolomic analysis was demonstrated to be possible by employing different methods, such as proton NMR (^1^H-NMR) or mass spectrometry (MS)^15,20–22^, which also highlighted that the metabolic changes can be detected in different biological samples, including urine, blood or cerebrospinal fluid (CSF)^23,24^. The separation of BD subjects treated with SGAs, BD subjects treated with lithium, and SCZ subjects treated with SGAs was possible by employing serum metabolomic studies, suggesting also the specificity of the pharmacometabolome to assist the diagnosis in clinics^25,26^. In addition, a set of metabolites that allowed the separation of BD and SCZ patients from HCs, and that could discriminate SCZ from BD and crack users, were recently identified by us and others^15,20,25–28^. These studies may represent a good example of how the metabolomic studies can bring new information about pathways and metabolic alterations, which might not be detectable by genetic studies. However, in these studies, the pathways identified for each individual metabolite could not be clearly associated with the etiology or pathophysiology of SCZ and BD.

In the current study, we applied a systems level approach by exploring together the altered metabolites in SCZ and BD, aiming to have new insights into the underlying pathophysiology of these MDs. A systems level approach is likely to compensate some missing information and discard noisy data from the process. For this purpose, we identified the genes/proteins that are closely related with the biomarker metabolites and which are connected through reliable protein–protein interactions (PPIs). After constructing a PPI network related to SCZ and BD, we applied a graph-clustering algorithm DPClusO to determine the clusters in the network. Then, we utilized the statistically significant clusters to identify important and common pathways underlying SCZ and BD.

## Methods and results

**Figure 1:**
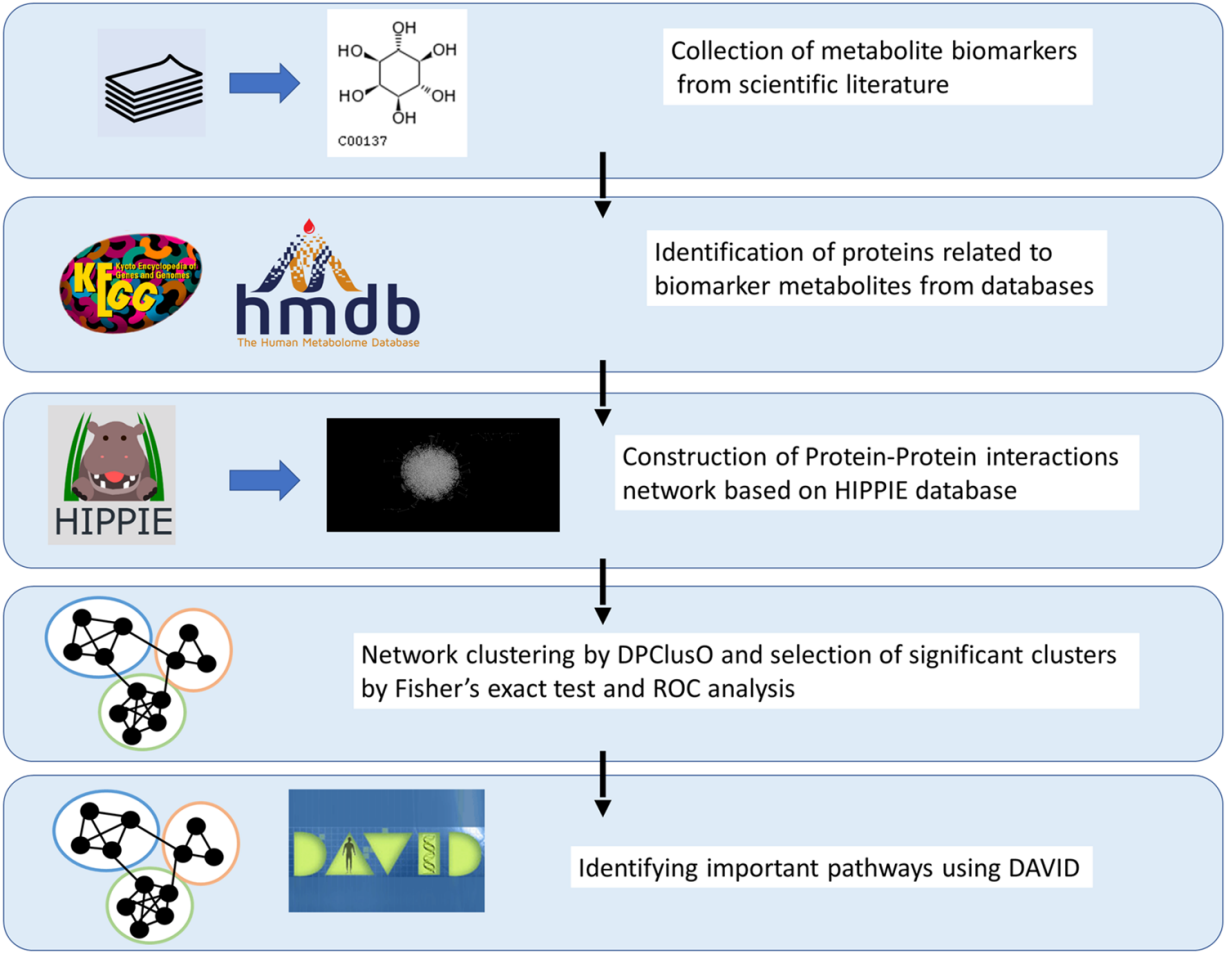
Flowchart showing each steps of the present research**.** The flowchart shows the analysis performed in the present work, starting from fishing out the most relevant metabolite biomarkers from online databases, such as MEDLINE/PubMed (maintained by The United States National Library of Medicine at the National Institutes of Health). Then, the analysis was performed using Kyoto encyclopedia of genes and genomes (KEGG), human metabolome database (HMDB), Human integrated protein–protein interaction rEference (HIPPIE), DAVID (The database for annotation, visualization and integrated discovery) and a network clustering algorithm (DPClusO).

The approach adopted in the present work is illustrated in the flowchart (Fig. 1), consisting of five major steps. The methods and corresponding results obtained in each step of the flowchart are discussed below in five separate sections, according to the flow order.

### Data collection of biomarkers

**Figure 2:**
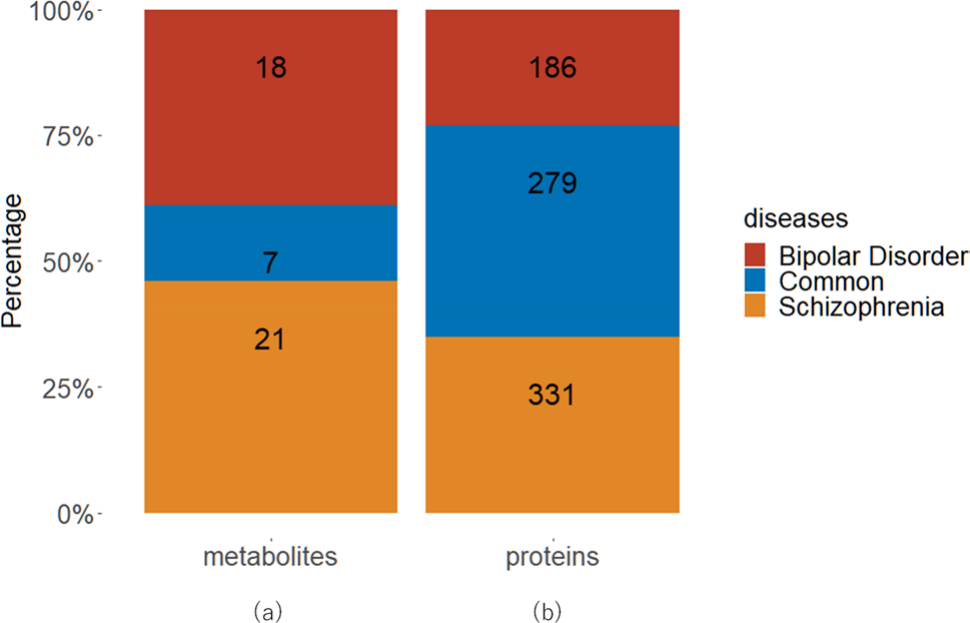
Proportion of unique and common (**a**) metabolites, (**b**) proteins regarding schizophrenia (SCZ) and bipolar disorder (BD). The metabolites and proteins present in BD, SCZ and common to both diseases are indicated by red, orange and blue colors respectively.

Biomarker metabolites were collected by searching the most relevant papers related to the target diseases, namely SCZ and/or BD. For this present study, we collected a total of 46 biomarkers, from which 28 were related to SCZ, 25 were related to BD and 7 were common to these both MDs. The proportions of SCZ and BD unique and common metabolites are shown in Fig. 2a.

These biomarkers were mostly detected by NMR, MS, or ion cyclotron type of experiments by using human biological samples, such as serum, blood etc. The collection of these two sets consisting of 46 biomarker metabolites includes: acetate, N-acetyl-d-mannosamine, 2,3-diphospho-d-glyceric-acid, alpha-ketoglutaric-acid, N-acetyl-l-alanine, arginine, choline, formate, glutamate, amygdalin, isocitric acid, myo-inositol, N-acetyl-glutamic-acid, phenylalanine, propionate, pyruvate, serine, beta-alanine, N-acetyl-l-phenyl-alanine, lipoamide, alpha-ketoisovaleric acid (MOA)*, l-glutamine, acetate, N-acetyl aspartyl-glutamic acid (NAAG), lactate, phosphocholine, alanine, citrate, cystine, eicosanoic acid, glucose, glycerate, β-hydroxybutyrate, pyroglutamic acid, sorbitol, taurine, tocopherol-alpha, uridine, l-threonine, adenine, glycine, adenosine, GABA, mannitol, pantothenate, 3-methyl-2-oxobutinoic acid, guanine. We added these data to KNApSAcK biomarker database in which the biomarkers of many other diseases have also been collected^29–31^.

### Collection of related proteins

**Figure 3:**
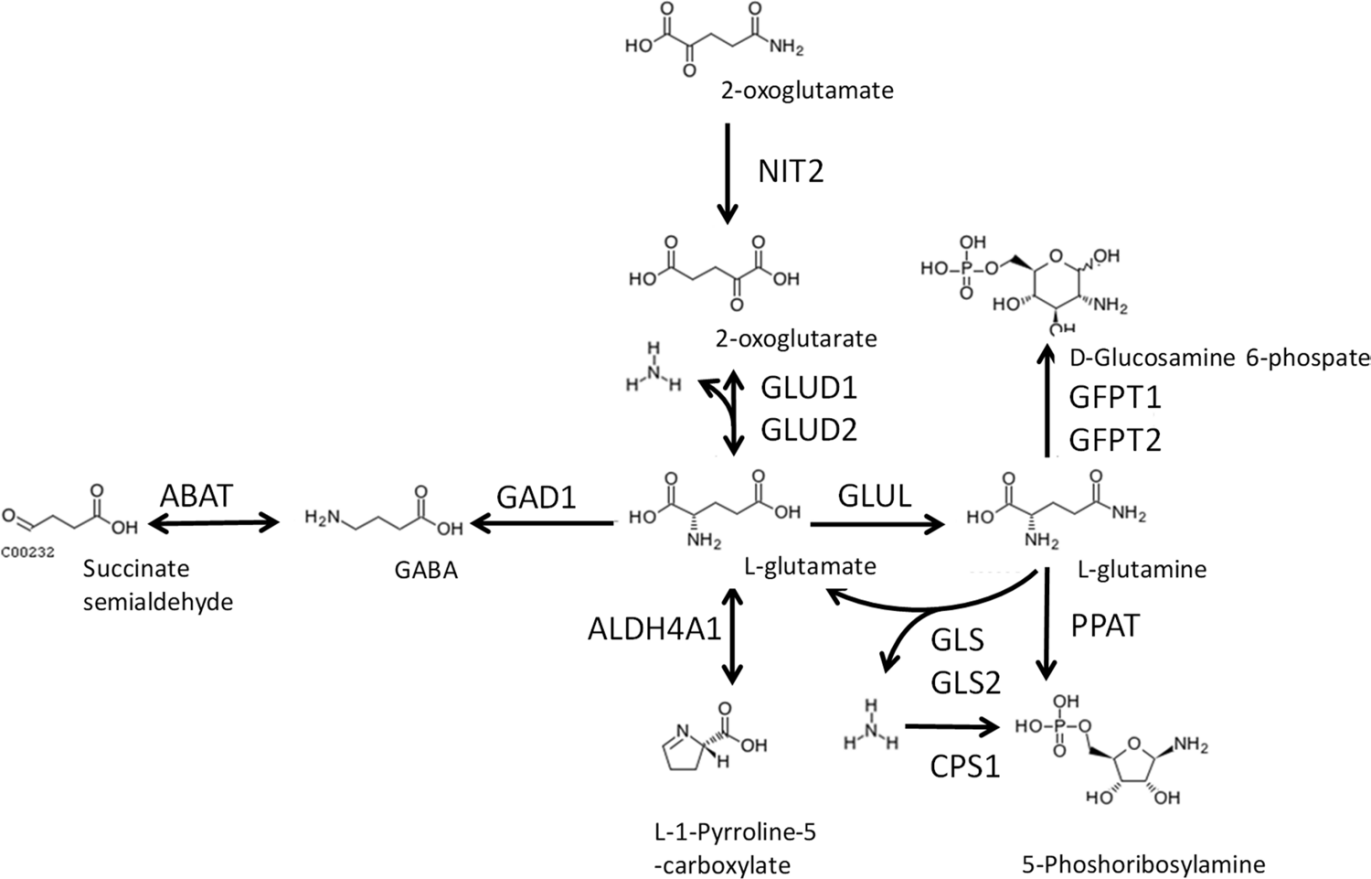
Partial l-glutamate pathway. *GLUD1* glutamate dehydrogenase 1, *GLUD2* glutamate dehydrogenase 2, *NIT2* omega-amidase NIT2, *ALDH4A1* aldehyde dehydrogenase 4 family member A1, *ABAT* 4-aminobutyrate aminotransferase, *GFPT1* glutamine-fructose-6-phosphate transaminase 1,*GFPT2* glutamine-fructose-6-phosphate transaminase 2, *GLUL* glutamine synthetase, *GLS* glutaminase, *GLS2* glutaminase 2, *PPAT* phosphoribosyl pyrophosphate amidotransferase, *GAD1* glutamate decarboxylase 1, *CPS1* carbamoyl-phosphate synthase 1, *GABA* gamma-aminobutyric acid.

We adopted two methods to collect SCZ and BD proteins related to the biomarkers reported for these MDs. Firstly, we employed the R package hmdbQuery^32^, which can provide pairwise associations between the metabolites and genes/proteins based on Human Metabolome Database (HMDB)^33^. Secondly, we utilized the metabolic pathway maps of the Kyoto Encyclopedia of Genes and Genomes (KEGG) database^34^. The enzymes up to the path length of two, from each target biomarker, were selected. Here, we illustrate the process using an example. Figure 3 shows a part of the KEGG pathway (human) concerning l-glutamate, which is the synonym for the biomarker Glutamate with KEGG ID C00025. In Fig. 3, we can see 4 branches connected to l-glutamate. All enzymes up to the path length of two, which include GLUD1, GLUD2, NIT2, ALDH4A1, ABAT, GFPT1, GFPT2, GLUL, GLS, GLS2, PPAT and GAD1, were all considered in our study. However, as for instance, CPS wasn’t selected, because it was considered out of the proposed protocol, as it was beyond the path length of two. We repeated this process for the 46 different biomarkers considered for this study (as listed above), and then we finally constructed the collection of these 46 sets of enzymes/proteins. Thus, we identified 331 + 279 = 610 and 186 + 279 = 495 proteins related to SCZ and BD, respectively, from which 279 proteins were common to both diseases (as shown in Fig. 2b, also possibly implying that both diseases are closely related. Therefore, for the sake of the system level analysis, it was reasonable to consider these two diseases together for the pathway analysis. Such integrated analysis is likely to allow the identification of common and other novel significant pathways for these two diseases.

### Construction of PPI network relevant to disease

SCZ and BD related PPI network was constructed using the human integrated protein–protein interaction rEference (HIPPIE) database, in which each interaction is characterized by a score value. The collection of proteins sets identified and depicted in Fig. 2b was considered as S. We selected all interactions regardless of the score value between proteins *a* and *b*, as such that *a* ∈ S and *b* ∈ S. In addition, we selected the interactions with score value > 0.7 between proteins *a* and *b*, as such that *a* ∈ S and *b* ∉ S. Then, we identified 3,233 interactions for BD, 4266 interactions for SCZ, and 1904 common interactions for SCZ and BD. In total, these 5595 interactions among a total of 3184 proteins composed our disease related PPI network. Additionally, in order to inspect the global network properties of this network, we employed Cytoscape tool, which is an open-source tool for visualizing and analyzing networks^35^. The degree distribution of this network is of power-law type. Other global topological properties of this network are as follow: clustering coefficient is 0.066, characteristic path length is 4.973, and diameter is 12. Networks with power law degree distribution are so called scale-free networks^36^. Scale-free networks are also small-world networks, if their average path length is small, and if diameter increases logarithmically with the number of vertices^37^. Therefore, our network consisting of 3184 nodes with a power law degree distribution, average path length 4.973 and diameter 12, can be considered as a small world network. These properties are consistent with a PPI network in general.

### Network clustering and selection of significant clusters

After constructing the disease relevant PPI network, clustering was performed using the DPclusO algorithm^38–41^. The DPClusO algorithm generates overlapping clusters and ensures coverage, i.e. each node goes to at least one cluster. We hypothesize that clustering of a disease relevant PPI network may help the isolation of systems with disease-related properties. Therefore, statistically significant PPI clusters that are enriched for SCZ and BD related proteins could be used to predict novel genes and pathways. Clustering was performed 9 times using input densities as 0.1, 0.2,…. up to 0.9. Table 1 shows the statistics data, *i.e*., the number of clusters, size of the biggest cluster, average cluster size and the number of significant clusters corresponding to 9 sets of the generated clusters. As expected, smaller density values resulted in larger and fewer number of clusters. The enrichment of proteins related to SCZ and BD was assessed in each identified cluster by Fisher’s exact test p-values. Additionally, p-values were corrected by Bonferroni and Hochberg False Discovery Rate (FDR).

**Table 1:** Results of each DPclusO clustering.

Density	Total clusters	Max size	Average size	Significant clusters
0.1	315	72	17.59683	75
0.2	531	38	10.00188	56
0.3	816	25	6.530637	33
0.4	1053	17	5.361823	29
0.5	1382	13	4.293054	25
0.6	1841	11	3.260185	2
0.7	2724	8	2.104993	49
0.8	2744	7	2.097303	43
0.9	2764	7	2.068017	61

With 9 different input densities, DPClusO generated 9 sets of clusters. In other words, the disease relevant PPI network was divided into clusters in 9 different ways. To assess which set of clusters was more useful for our purpose, we performed a receiver-operating characteristic (ROC) analysis. For ROC analysis, we assigned the SScore (Significance Score)^42^, to each gene, based on the *p*-values of the clusters to which they belong to. SScore of a protein is defined as following:

$$ {\text{SScore}} =  - \log (FDR). $$

We assigned a SScore value to each protein. However, as DPclusO performs overlapping clustering, each protein may have more than one SScores, from which we considered only the highest SScore for the ROC analysis. SCZ and BD related genes/proteins were downloaded from the DisGeNet database, and we considered the set collection of DisGeNet data and the extracted set of SCZ and BD related proteins as the true positive proteins related to these MDs. The ROC curve was created by selecting a series of threshold SScore values to generate the true positive rates (TPR) and false positive rates (FPR).

**Figure 4:**
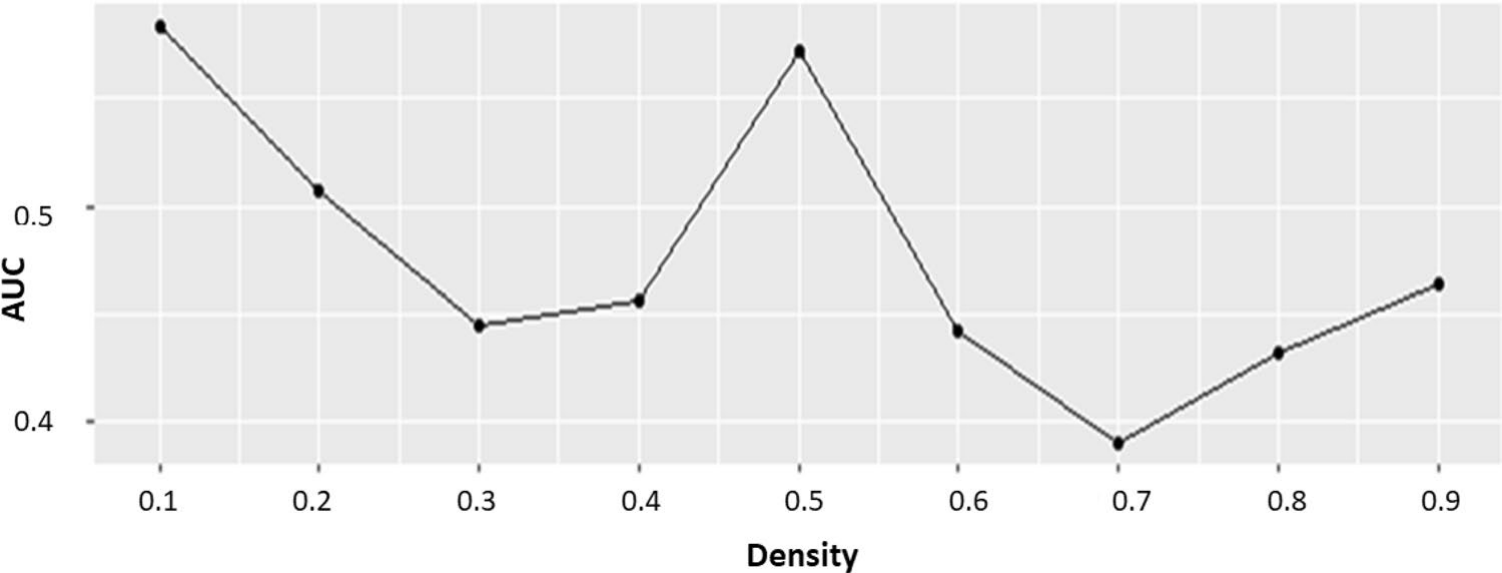
The area under the curve (AUC) values for 9 different input densities used for clustering by DPClusO.

The area under the curve (AUC) values corresponding to different densities are shown in Fig. 4. The highest AUC value was obtained for density 0.1. Then, we employed the clusters generated using density 0.1 for the pathway analysis. Some clusters included non-MD proteins together with currently known MD proteins.

### Identification of SCZ and BD related pathways

**Figure 5:**
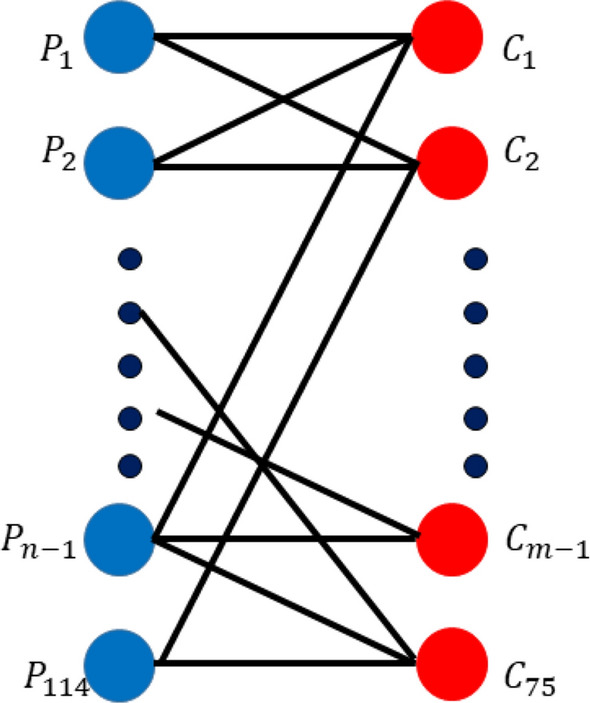
Bipartite graph linking significant clusters and corresponding significant pathways.

For pathway analysis, we employed the online resources from the Database for Annotation, Visualization and Integrated Discovery (DAVID)^43^, in which a list of genes is considered to conceive the biological meaning for a gene group. In the previous section, based on the highest AUC value we selected 75 statistically significant clusters with FDR < 0.05. We collected the Uniprot_Accession_IDs for the genes, representing the proteins included in those 75 significant clusters. We entered each of these 75 clusters to DAVID separately, and significantly associated KEGG pathways based on count = 2, EASE score = 0.1 and FDR ≤ 0.05 were retrieved. Count is the number of common genes/proteins between an input set and a pathway. EASE is the modified Fisher’s Exact *p*-value. DAVID Function Annotation tool applies Fisher’s exact test and provides a *p*-value for each pathway indicating its significance. For each cluster we have chosen the top 3 significant pathways. The significant PPI clusters can be considered as sub-systems relevant to SCZ and BD. We hypothesize that the pathways associated to many significant clusters are more relevant to SCZ and BD. Therefore, we finally made a bipartite graph, as shown in Fig. 5, linking these 75 clusters (one set of nodes) with the union of all significant pathways (another set of nodes) chosen for these 75 clusters. From the bipartite graph, 28 high degree pathways (degree ≥ 3) were selected as SCZ and BD related pathways.

**Figure 6:**
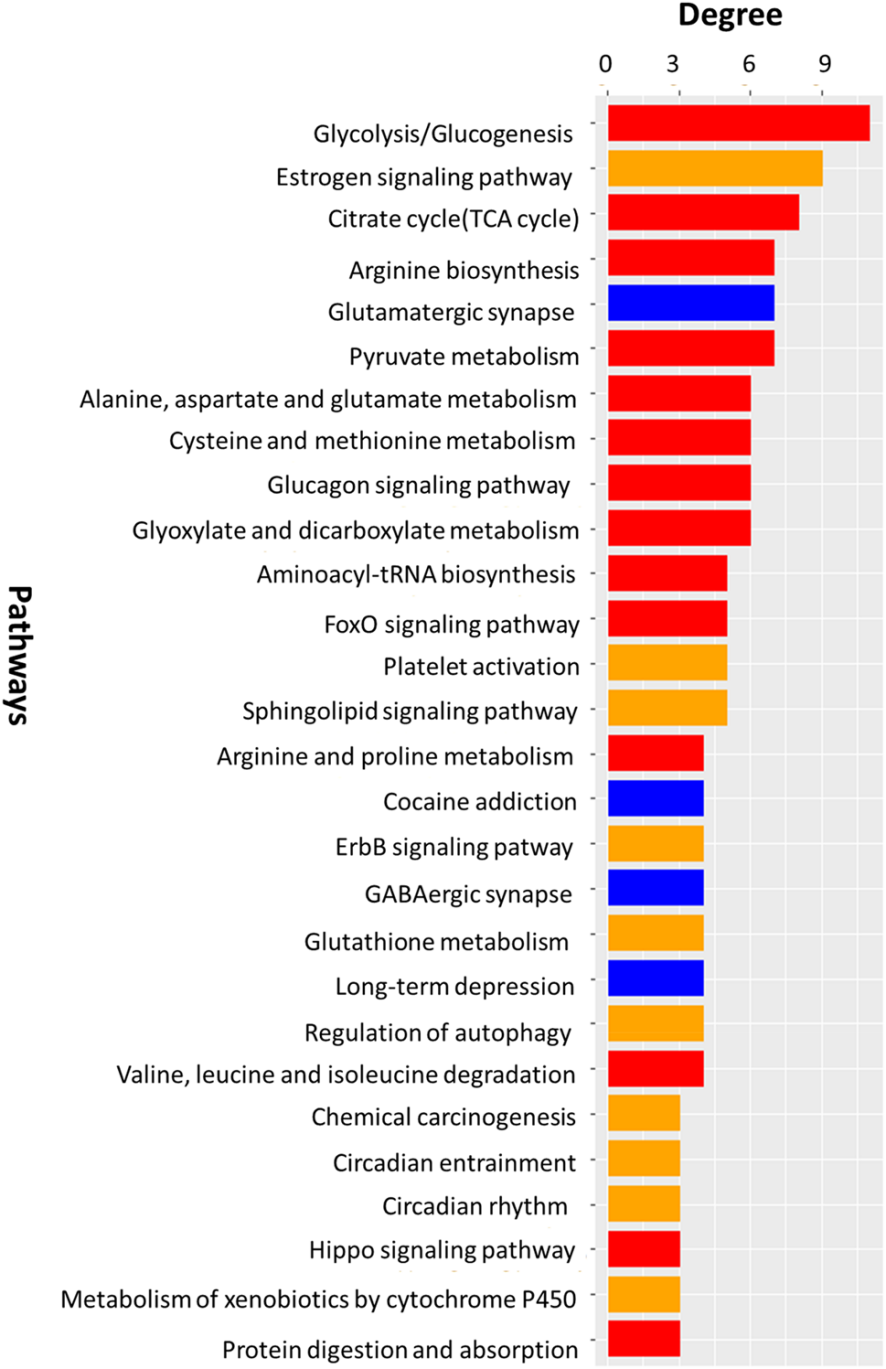
SCZ and BD related pathways predicted in this study (degree ≥ 3, for 28 pathways). Red, yellow and blue bars are pathways related to energy systems, stress response and neuron systems respectively.

These selected pathways are: glycolysis/gluconeogenesis, estrogen signaling pathway, citrate cycle (TCA cycle), arginine biosynthesis, glutamatergic synapse, pyruvate metabolism, alanine/aspartate/glutamate metabolism, cysteine and methionine metabolism, glucagon signaling pathway, glyoxylate and dicarboxylate metabolism, aminoacyl-t-RNA biosynthesis, FoxO signaling pathway, platelet activation, sphingolipid signaling pathway, arginine/proline metabolism, cocaine addiction, ErbB signaling pathway, GABAergic synapse, glutathione metabolism, long-term depression, regulation of autophagy, valine/leucine/isoleucine degradation, chemical carcinogenesis, circadian entrainment, circadian rhythm, hippo signaling pathway, metabolism of xenobiotics by cytochrome P450, and protein digestion and absorption. These 28 identified pathways and their respective degrees are depicted in Fig. 6, and according to the KEGG pathway database, they could be classified into three main groups, namely energy systems, stress response and neuron systems.

## Discussion

Due to the disparate and inconsistent findings from various biomarker studies in mental disorders (MDs), in the present work we aimed to identify the important pathways, predicted to be underlying SCZ and BD based on metabolite biomarkers. These 28 pathways identified herein are mainly involved in three major systems, namely stress response, energy and neuron systems, and they are all significantly related to SCZ and BD, as supported by several evidences reported in the literature and presented here in a simplified way as follow.

The majority of pathways identified in this study are associated with energy metabolism. In fact, among the identified SCZ and BD related pathways, those presenting the highest degree are the (1) *glycolysis/gluconeogenesis*; (2) *pyruvate* (which is the output of the metabolism of glucose) *metabolism*; (3) *glucagon* (which increases glycogenolysis and gluconeogenesis) *signaling pathway*; (4) *citrate cycle* (TCA cycle) (which is an important aerobic pathway for the final steps of the oxidation of carbohydrates and fatty acids); and (5) *FoxO signaling pathway* (which plays important roles in regulation of gluconeogenesis and glycogenolysis by insulin signaling and which affects many cellular physiological processes such as cell cycle, apoptosis, metabolism and oxidative stress, immune regulation). Interestingly, it is well accepted that patients with MDs often present metabolic vulnerabilities with consequent risk of developing cardiometabolic comorbidities^44^, representing one of the leading causes for premature death and reduced lifespan of patients with SCZ or BD^44,45^. In fact, several authors have suggested the involvement of abnormalities in energy metabolism and brain glucose utilization in the pathophysiology of these psychiatric disorders^44,45^. Moreover, proteomic studies with *post-mortem* brains have also provided evidences for energy metabolism dysfunction in several MDs^46^, as well as Schubert et al. reported an increased glycolysis in SCZ and BD^47^.

Moreover, multiple pieces of evidence have also suggested that brain energy metabolism, mitochondrial functions and redox balance are impaired to various degrees in BD and SCZ, and mitochondrial dysfunction and the resultant metabolic changes leading to oxidative stress may also be important etiological factors in the context of these MDs^48–51^.

Mitochondria have a central role in the energy metabolism, and implication of mitochondrial function alterations in the etiology of SCZ is recognized^52^. Furthermore, it is suggested that mitochondrial respiration is downregulated in depression, and upregulated during mania in BD, whilst in SCZ, the number of mitochondria and mitochondrial respiration are both downregulated^53^. Moreover, the mitochondrial dysfunction in blood platelets of patients with manic episodes was proposed as a ‘trait’ marker of BD^54^. One of our identified pathways is *platelet activation*. At this point, it is also worth mentioning that lithium is also key to a wide range of processes at all levels, from neuroprotection to oxidative stress and energy production^55^. In addition, lithium has unquestionable therapeutic superiority for BD treatment, while it also plays an important role in mitochondrial function, which is improved via its role in phospholipid metabolism and inositol depletion^55^.

Possibly as a response to these abnormalities in metabolism and oxidative stress, several compensatory pathways were also identified in the present study, as for instance, the (1) *glutathione metabolism*, which plays important roles in antioxidant defense and its deficiency contributes to oxidative stress; (2) *sphingolipid signaling pathway* which regulates cellular responses to stress; and (3) *ErbB signaling pathway* which regulates diverse biologic responses, including cell proliferation and survival, and regulation of autophagy that is involved in cell growth, survival, development and death, and linked to neurodegeneration in many other disorders, besides being a stress-induced catabolic process. In fact, the main biological alterations of BD and SCZ pertain to inflammation, oxidative stress, membrane ion channels, metabolic dysfunction and circadian system^56–59^. Interestingly, we have also identified here the circadian entrainment and circadian rhythm as highly important pathways associated with the metabolites found in BD and SCZ.

Energy pathways and synaptic function were also implicated in neuropsychiatric disorders such as SCZ and BD^60^. Among the pathways related to neuron systems found in the present work are the (1) *glutamatergic synapse,* (2) *GABAergic synapses,* (3) *long-term depression (LTD),* (4) *cocaine addiction*, amongst others (as shown in Fig. 6). These pathways are implicated in synaptic/neuronal differentiation, plasticity, and migration, as well as the activation of metabotropic glutamate receptors in the prefrontal cortex induces LTD and reduces the stress-induced anhedonia and other stress-related behavioral impairments in MDs^61^. The LTD is a type of synaptic plasticity in which the efficacy of signal transmission across a synapse continuously decreases after a certain triggering activity, and this activity-dependent plasticity may also result in a persistent enhancement of synaptic transmission^62^. While the LTD pathway has F ≥ 35, the long-term potentiation pathway has F < 20, therefore, suggesting together that LTD may be implicated in the cognitive dysfunctions observed in SCZ and BD, although not demonstrated or explored in clinics up to now.

We have also identified pathways related to several amino acids metabolism and synthesis (e.g. alanine/aspartate/glutamate metabolism, cysteine and methionine metabolism, arginine/proline metabolism, arginine biosynthesis etc.) and *protein digestion/absorption* pathway, which are important not only for the protein biosynthesis, but also for the functions interrelated with glucose metabolism, synthesis of neurotransmitters, and production of energy, whilst some of them have also the ability to modulate the inflammatory and immune systems. The association between MDs and inflammation/neuroinflammation has been widely discussed and accepted by many, and the correlation of pro-inflammatory markers with symptoms intensity was also reported in SCZ and BD^63^. Two other identified pathways are *Hippo Signaling pathway* and *metabolism of xenobiotics by cytochrome p450*. Cytochrome P450 enzymes (CYPs) play a crucial role in metabolism of xenobiotics in human brain. Recent advances support role of these enzymes in the pathogenesis of psychiatric and neurodegenerative disorders such as depression, and schizophrenia^64^. Hippo Signaling pathway is known to have involvement in stress-related psychiatric disorders^65^.

Taken together, all presented data are in good agreement with the theoretical framework for metabolic comorbidities of mood disorders in which immune system has been likewise “selfish” due to independent energy consumption, which may compete with the brain (another high energy-consumer) for glucose, which may explain the various conditions of medical impairment, as the Metabolic Syndrome (MetS), obesity, type 2 diabetes mellitus (T2DM) and immune dysregulation, often reported in neuropsychiatric patients^66^.

In addition, some other unexpected pathways were also identified here, as the *estrogen signaling pathway*. This pathway is frequently associated with the activation of various protein-kinase cascades, and *aminoacyl-t-RNA biosynthesis*, which play a central role in protein biosynthesis. Certainly, these protein-kinase cascades and protein biosynthesis deserve special attention. Further studies of these pathways in the context of BD and/or SCZ may have the power to bring new insights into these MDs.

Lastly, good biomarkers for early diagnosis could allow clinical early diagnosis and possibly more adequate treatment^67^. Therefore, early diagnosis of SCZ and BD would be essential to improve outcomes, as early intervention was found to be beneficial for the patients to prevent the cognitive deficits and disabilities if early and properly treated with appropriate pharmacotherapy^68–70^. However, as most of them could not be replicated, unfortunately, no biomarker has been established for differentiating BD and HCs until present^71^, with exception to our most recently work describing a set of metabolic peripheral biomarkers that allow the differential diagnosis between SCZ and BD^20^. However, the absence of any conclusive evidence to identify these severe mental illnesses at an early stage, based on biomarkers alone, led us to propose the integration of these metabolome data by using system biology approach based on protein–protein interactions for identifying pathways underlying SCZ and BD pathophysiology, as presented here. The possible inclusion of some of these more reliable biomarkers, in a study conducted as presented here, may also further increase the power of bringing even more valuable insights into the actual knowledge of pathways underlying these diseases, possibly contributing to early diagnosis and/or a better clinical management.

## Conclusion

In this work we presented a method for identifying pathways underlying Schizophrenia and Bipolar Disorder based on potential biomarkers and PPI network. This study started by collecting a list of potential biomarkers and only those genes that have strong links with the biomarkers and that are connected via reliable PPIs were involved. After constructing a PPI network linked exclusively to SCZ and BD, we identified 75 statistically significant clusters. Based on these clusters, we identified 28 significant pathways, suggesting that SCZ and BD onset may be mainly associated with the abnormality of energy systems, and neuron functions and stress response, which were shown by many to be affected in these MDs. Our results also support the mitochondrial hypotheses for these mental disorders (MDs), and further studies targeting mitochondria function and long-term depression (LTD) may have the power to strongly contribute for the understanding of the mechanism underlying these MDs. Novel pathways, never associated with MDs previously, were also identified here, such as the protein-kinase cascades, LTD and protein biosynthesis. These pathways certainly deserve further attention and studies, aiming to aid in the diagnosis and/or clinical management of MDs. More importantly, we highlight that the present proposed method for finding disease pathways, starting from metabolite biomarkers, is potentially applicable for any other disease.

## Data Availability

Dataset may be sent upon request.
